# Extremely short duration interval exercise improves 24-h glycaemia in men with type 2 diabetes

**DOI:** 10.1007/s00421-018-3980-2

**Published:** 2018-08-31

**Authors:** Richard S. Metcalfe, Ben Fitzpatrick, Sinead Fitzpatrick, Gary McDermott, Noel Brick, Conor McClean, Gareth W. Davison

**Affiliations:** 1 Applied Sports Technology, Exercise and Medicine Research Centre (A-STEM), Swansea University, Bay Campus, Fabian Way, Swansea, SA1 8EN UK; 2 Sport and Exercise Science Research Institute, Ulster University, Belfast, Northern Ireland UK; 3 Western Health and Social Care Trust, Altnagelvin Area Hospital, Derry, Northern Ireland UK; 4 School of Sport, Ulster University, Belfast, Northern Ireland UK; 5 Psychology Research Institute, Ulster University, Belfast, Northern Ireland UK

**Keywords:** Exercise, High-intensity interval training, Postprandial glucose, Continuous glucose monitoring, Type 2 diabetes

## Abstract

**Purpose:**

Reduced-exertion high-intensity interval training (REHIT) is a genuinely time-efficient exercise intervention that improves aerobic capacity and blood pressure in men with type 2 diabetes. However, the acute effects of REHIT on 24-h glycaemia have not been examined.

**Methods:**

11 men with type 2 diabetes (mean ± SD: age, 52 ± 6 years; BMI, 29.7 ± 3.1 kg/m^2^; HbA_1c_, 7.0 ± 0.8%) participated in a randomised, four-trial crossover study, with continual interstitial glucose measurements captured during a 24-h dietary-standardised period following either (1) no exercise (CON); (2) 30 min of continuous exercise (MICT); (3) 10 × 1 min at ~ 90 HR_max_ (HIIT; time commitment, ~ 25 min); and (4) 2 × 20 s ‘all-out’ sprints (REHIT; time commitment, 10 min).

**Results:**

Compared to CON, mean 24-h glucose was lower following REHIT (mean ± 95%CI: − 0.58 ± 0.41 mmol/L, *p* = 0.008, *d* = 0.55) and tended to be lower with MICT (− 0.37 ± 0.41 mmol/L, *p* = 0.08, *d* = 0.35), but was not significantly altered following HIIT (− 0.37 ± 0.59 mmol/L, *p* = 0.31, *d* = 0.35). This seemed to be largely driven by a lower glycaemic response (area under the curve) to dinner following both REHIT and MICT (− 11%, *p* < 0.05 and *d* > 0.9 for both) but not HIIT (− 4%, *p* = 0.22, *d* = 0.38). Time in hyperglycaemia appeared to be reduced with all three exercise conditions compared with CON (REHIT: − 112 ± 63 min, *p* = 0.002, *d* = 0.50; MICT: -115 ± 127 min, *p* = 0.08, *d* = 0.50; HIIT − 125 ± 122 min, *p* = 0.04, *d* = 0.54), whilst indices of glycaemic variability were not significantly altered.

**Conclusion:**

REHIT may offer a genuinely time-efficient exercise option for improving 24-h glycaemia in men with type 2 diabetes and warrants further study.

## Introduction

Exaggerated postprandial glycaemic excursions are strongly correlated with type 2 diabetes-related complications, including cardiovascular disease, which is a major cause of morbidity and mortality (Monnier and Colette 2015). However, studies employing continuous glucose monitoring (CGM) have shown that, despite pharmacological intervention, a large proportion of patients with type 2 diabetes (and even those well controlled according to HbA_1c_) still spend significant portions of the day in hyperglycaemia (van Dijk et al. 2011). This emphasises the importance of a multi-component treatment approach in type 2 diabetes, incorporating regular exercise, which is an effective strategy for lowering postprandial glucose excursions (Van Dijk et al. 2013) and HbA_1c_ (Church et al. 2010) over and above improvements seen with first-line drug therapies. Whilst exercise training-induced adaptations may result in an additive and more prolonged improvement in insulin sensitivity (i.e. > 72 h post-training) (Dela et al. 1995), it is generally accepted that the cumulative impact of regular (i.e. daily) acute exercise on glycaemic control (van Dijk et al. 2012) is of greater clinical importance for the long-term management of glycaemic control in type 2 diabetes (Colberg et al. 2016).

The exercise recommendations for individuals with type 2 diabetes are similar as for the general population, suggesting a minimum of 150 min of moderate–vigorous-intensity aerobic exercise each week (Colberg et al. 2016; Garber et al. 2011). However, self-report data suggest that adherence to these guidelines is poor in the general population (Allender et al. 2008; Hallal et al. 2012) and potentially even lower in those with type 2 diabetes (Morrato et al. 2007). The reasons for poor exercise adherence are numerous and complex, but a perceived lack of time is consistently reported as one of the important barriers in people with type 2 diabetes (Korkiakangas et al. 2009). In response to this, submaximal high-intensity interval training (HIIT) and supramaximal sprint interval training (SIT) have been proposed as time-efficient alternative exercise options for improving glycaemic control. Acute studies in overweight individuals (Little et al. 2014) and people with type 2 diabetes (Terada et al. 2016) have shown superior improvements in glycaemic control with HIIT compared with 30–60 min of traditional MICT. Despite the case for a superior clinical benefit, the total time commitment, including recovery intervals, means that most HIIT protocols are not as time-efficient as often claimed. To date, the protocols studied generally require 20–60 min (Terada et al. 2016; Little et al. 2011; Gillen et al. 2012), which is no different (and in some cases, exceeds) than current exercise recommendations for MICT (Colberg et al. 2016; Garber et al. 2011). Moreover, there is currently vigorous debate about whether either HIIT or SIT would be appropriate exercise strategies for recommendation to the general population or specific patient populations, based on the hypothesised potential for ‘unpleasant’ perceptual responses (e.g. high perceived exertion and negative affect) to lead to low exercise adherence (Hardcastle et al. 2014). The total time commitment and potential for unpleasant perceptual responses increase as a function of the number and duration of high-intensity efforts (Townsend et al. 2017). Thus, it is important to examine whether HIIT/SIT protocols, with fewer and shorter high-intensity efforts, remain efficacious for improving insulin sensitivity and glycaemic control in type 2 diabetes (Vollaard and Metcalfe 2017).

There is evidence that HIIT/SIT protocols with fewer and/or shorter sprints can improve glucose tolerance in healthy sedentary individuals (Metcalfe et al. 2012, 2016; Gillen et al. 2016). For example, we previously demonstrated that a modified SIT intervention, consisting of 10 min of low-intensity cycling interspersed with two 20 s ‘all-out’ sprints [termed ‘reduced-exertion high-intensity interval training’ (REHIT)], was effective at improving insulin sensitivity in sedentary men over 6 weeks (Metcalfe et al. 2012). Importantly, these benefits were observed despite the low time commitment (10 min per session) and acceptable session ratings of perceived exertion (‘somewhat hard’). Ruffino et al. (2017) recently applied REHIT in type 2 diabetes and observed superior improvements in aerobic capacity and similar changes in resting blood pressure compared with a moderate-intensity walking intervention over an 8-week training period. REHIT did not improve insulin sensitivity or reduce 24-h mean glucose in this study, however, responses were captured 3 days following training cessation and improvements may have been lost at this time point (Ruffino et al. 2017). Nevertheless, 8 weeks of REHIT did lower plasma frucotosamine concentrations (a marker of average blood glucose levels in the preceding 2–4-week period), suggesting improved glycaemic control during the training intervention (Ruffino et al. 2017). Yet, the acute effects of REHIT on post-exercise glycaemic control in people with type 2 diabetes have not been explored. Thus, the primary aim of this study was to examine the effect of a single bout of REHIT on 24-h glycaemia in men with type 2 diabetes relative to a no-exercise control trial using continuous glucose monitors. Our primary hypothesis was that REHIT would improve glycaemic control relative to no exercise. We also studied the effects of a single bout of MICT and a single bout of HIIT compared with no exercise, as both have previously been shown to improve glycaemic control (Gillen et al. 2012; van Dijk et al. 2012).

## Methods

### Ethical approval

This randomised-controlled acute crossover trial was conducted at Ulster University, Northern Ireland (UK), between October 2016 and August 2017 (ClinicalTrials.gov registration: NCT03082859). The experimental protocol was approved by the Office for Research Ethics in Northern Ireland (RECA ref: 16/NI/0115) and conducted in accordance with the Declaration of Helsinki.

### Participants

**Fig. 1:**
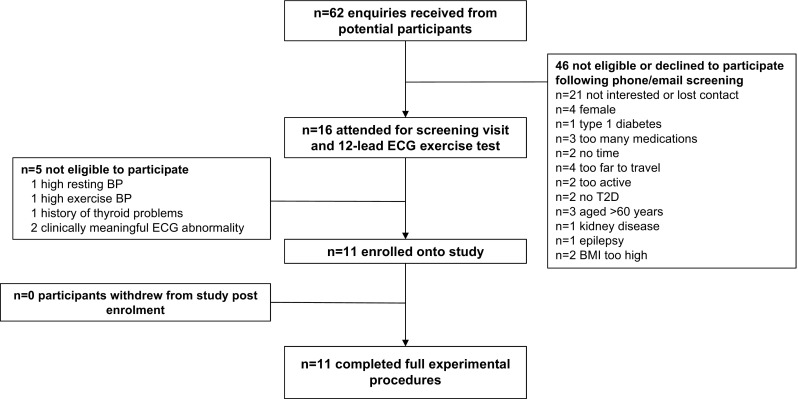
Flow of participants through the study

11 (*n* = *11*) men, diagnosed with type 2 diabetes mellitus by a clinician 4 ± 3 (range 0.5–8) years previously, completed the full experimental procedures (Fig. 1; Table 1). Exclusion criteria included exogenous insulin therapy, currently taking more than two glucose-lowering medications, BMI ≥ 40 kg/m^2^, classification as highly active on the international physical activity questionnaire (IPAQ) (Craig et al. 2003), and any contraindications to exercise, including any history of cardiovascular or cerebrovascular disease, impaired renal or liver function, and hypertension not well controlled by standard medication. All participants were informed about the study, both verbally and in writing, before providing their written consent to participate. Eligible participants completed a 12-lead exercise stress test on a cycle ergometer (Lode Corival; Lode, Groningen, Netherlands) and received clearance for vigorous-intensity exercise from a clinical cardiac physiologist. At baseline, all medication was recorded and participants were instructed to maintain their usual dose/timing/type of medication throughout the study period.

**Table 1:** Participant characteristics (*n* = 11)

Characteristics	Mean ± SD (range)
Age (years)	52 ± 6 (40–60)
Body mass (kg)	91.8 ± 10.4 (65.6-100.9)
Height (m)	1.76 ± 0.07 (1.66–1.89)
BMI (kg/m^2^)	29.7 ± 3.1 (23.8–34.1)
*V̇*O_2max_ (ml/kg/min)	28.9 ± 4.8 (18.3–35.3)
Systolic blood pressure (mmHg)	125 ± 12 (114–148)
Diastolic blood pressure (mmHg)	78 ± 7 (70–88)
Mean arterial pressure	93 ± 8 (83–108)
Duration of T2D (years)	4 ± 3 (0.5-8)
Fasting glucose (mmol/l)	8.1 ± 1.2 (7.1–9.7)
HbA_1c_ (mmol/mol)	52 ± 9 (38–67)
HbA_1c_ (%)	7.0 ± 0.8 (5.6–8.3)
Medication (*n*)	
Metformin	7
Metformin + sulfonylurea	4
Blood pressure lowering	1
Statin	5

*T2D* type 2 diabetes

### Pre-experimental procedures

Following health screening, participants completed a maximal incremental cycling test to volitional exhaustion to determine peak oxygen uptake (*V̇*O_2peak_), peak power output (*W*_max_) and peak heart rate (HR_max_). Following a 5-min warm up at 50 W, the intensity was increased by 15 W/min until cadence could not be maintained at ≥ 50 rpm (Lode Corival). *V̇*O_2peak_ was determined as the highest ten-breath rolling average, *V̇*O_2_ measured using an online gas analysis system (Cosmed Quark; CPET, Rome, Italy), and accepted if two or more of the following criteria were met: (1) volitional exhaustion, (2) a plateau in *V̇*O_2_ despite increasing intensity, (3) RER > 1.15, and (4) maximal heart rate within 10 beats of the age-predicted maximum (i.e. 220—age) (Howley et al. 1995). This was the case for all participants. Participants also completed two familiarisation sessions, on separate days, each lasting approximately 20 min, to introduce participants to the technique and effort required to perform all-out cycling sprints.

### Main experimental protocol

Each participant completed four main experimental trials (CON, REHIT, HIIT and MICT) in a randomised order (envelope method), with each trial taking place over 3 days. During each trial, participants underwent 24 h of continuous glucose monitoring under standardised dietary intake and the following experimental conditions: (1) a no-exercise control condition (CON); (2) a single bout of high-intensity interval training (HIIT); (3) a single bout of reduced-exertion high-intensity interval training (REHIT); and (4) moderate–vigorous-intensity continuous exercise (MICT). Each trial was separated by at least 5 days, and prior to each trial, participants were asked to avoid any structured exercise for 2 days. This was confirmed via physical activity monitoring using synchronised accelerometry and heart rate monitoring with branched model equations (Actiheart, Cambridge Neurotechnology Ltd., Cambridge, UK). The Actiheart is a non-invasive physical activity monitor that is both reliable and valid, can accurately estimate energy expenditure across low-, moderate- and high-intensity physical activities, and provides useful quantitative data on patterns of physical activity, allowing a comprehensive characterisation of physical activity status (Thompson et al. 2006). Participants wore the monitor continuously (day and night) and were instructed to only remove it when showering or bathing. There were no differences in physical activity across the 2 days prior to each experimental trial (*p* > 0.05 for all main effects of condition, respectively; Table 2). The Actiheart monitors were also worn throughout each main 24-h monitoring period.

**Table 2:** Dietary composition for the pre-trial evening meal and the main trial day

	Pre-trial evening meal (day 1)	Main trial day (day 2)
Total energy content	796 ± 187	2441 ± 235
CHO (kcal)	365 ± 79	1243 ± 100
Fat (kcal)	261 ± 99	761 ± 156
Pro (kcal)	171 ± 49	436 ± 49
CHO (%)	47 ± 8	51 ± 3
Fat (%)	32 ± 7	31 ± 4
Pro (%)	21 ± 4	18 ± 2
Breakfast (% of total energy)	–	20 ± 5
Lunch (% of total energy)	–	29 ± 4
Dinner (% of total energy)	–	30 ± 5
Snacks (% of total energy)	–	21 ± 6

Data shown as mean ± SD

*CHO* carbohydrate, *Pro* protein

Participants attended the lab between 15:00 and 18:00 h on day 1 for insertion of a subcutaneous glucose sensor (Enlite, Medtronic Inc., Minneapolis, USA) and CGM in the abdomen (iPro2, Medtronic Inc. Minneapolis, USA). Sensors were inserted approximately 5 cm from the umbilicus and on the opposite side from which participants tended to sleep on. The iPro2 measures glucose in the interstitial fluid every 5 min and values are subsequently converted to blood glucose using capillary measurements taken by each participant before each main meal and before sleep (Accu-Check; Roche Diagnostics, Basel, Switzerland). It has previously shown good validity and reliability when compared to blood glucose measured simultaneously from an intravenous cannula (Bailey et al. 2014). Participants then returned home but were provided with a standardised evening meal and snack (Table 2).

Participants returned to the laboratory the following morning (day 2) between 07:00 and 08:30 h to consume breakfast and complete the exercise session (30 min post-breakfast). During CON, participants remained sedentary throughout this entire period (i.e. ~ 2 h). Participants then returned to their normal daily routine but were provided with standardised meals (lunch, evening meal, snacks, to be consumed at standardised times (Table 2)). Participants returned to the laboratory the following morning for removal of the CGM.

### Dietary standardisation

Participants were provided with a standardised food and (energy containing) drink packages containing meals, snacks and drinks for each 42 h trial period (~ 18:00 h on day 1 to ~ 12:00 h on day 3; Table 3). The diet was designed to meet resting metabolic rate [determined using the Harris and Benedict equation (Harris and Benedict 1918)] multiplied by a PA level of 1.4. The macronutrient content was composed according to the 2008 ADA dietary recommendations for type 2 diabetes (Bantle et al. 2008) and consisted of three meals and snacks per full day. The contents of the dietary package were self-selected by participants (in consultation with the principle investigator) from a pre-determined list of foods available from a local supermarket. In this way, individual food preferences and tolerances were taken into account but the investigators were able to ensure appropriate energy and macronutrient content (Bantle et al. 2008). The food and drinks were ingested at pre-determined times for each participant so that a completely standardised diet was consumed during all four experimental trials. In addition to any energy-containing drinks provided to participants in food packages, participants were allowed to consume water ad libitum throughout each trial.

**Table 3:** Actiheart-derived physical activity energy expenditure prior to and during each experimental trial

	Pre-trial (2 days)				Main trial day			
	CON	REHIT	HIIT	MICT	CON	REHIT	HIIT	MICT
TEE (Kcal/day)	2869 ± 363	2847 ± 319	3000 ± 487	2889 ± 307	2719 ± 258	2913 ± 435	3325 ± 641*	3225 ± 356*
PAL	1.49 ± 0.18	1.48 ± 0.17	1.56 ± 0.24	1.51 ± 0.15	1.41 ± 0.12	1.51 ± 0.21	1.73 ± 0.32*	1.69 ± 0.16*
Sedentary time (mins)	1107 ± 119	1091 ± 139	1062 ± 155	1083 ± 132	1166 ± 91	1091 ± 120	1000 ± 146*	1006 ± 95*
Light PA (mins)	244 ± 84	271 ± 99	265 ± 108	263 ± 96	212 ± 62	257 ± 72*	282 ± 72*	294 ± 69*
Moderate PA (mins)	85 ± 55	77 ± 53	107 ± 77	93 ± 45	61 ± 43	87 ± 65	135 ± 96^	119 ± 52^
Vigorous PA (mins)	4 ± 11	1 ± 1	6 ± 14	1 ± 2	1 ± 1	5 ± 7	21 ± 16*	21 ± 24

Data shown are mean ± SD

*TEE* total energy expenditure, *PAL* physical activity level, *PA* physical activity

*p < 0.05 vs control

^*p* = 0.06 vs control

### Exercise bouts

#### REHIT

The REHIT bout was performed on a mechanically braked cycle ergometer (Monark Peak Bike; Monark, Vansbro, Sweden) and was based on the protocol used in previous studies (Metcalfe et al. 2012, 2015, 2016; Ruffino et al. 2017). It consisted of 10 min of unloaded pedalling interspersed with two ‘all-out’ sprints against a resistance equivalent to 5% of body mass. Just before each sprint, participants increased their pedal cadence to their maximal speed; the braking force was applied to the ergometer and participants maintained the highest possible cadence against the resistance for 20 s. Sprints were performed at 2 min 40 s and 6 min 40 s into the 10-min exercise session.

#### HIIT

The HIIT bout was performed on an electronically braked cycle ergometer (Lode Corival; Lode, Groningen, Netherlands) and involved 10 × 60 s cycling efforts interspersed with 60 s of low-intensity recovery (25 W) as previously described (Little et al. 2011; Gillen et al. 2012). Individual workloads were set at 85% *W*_max_, as pilot work has suggested this was appropriate for achieving ~ 90% maximal heart rate (HR_max_) during the final intervals.

#### MICT

MICT was performed on an electronically braked cycle ergometer (Lode Corival; Lode, Groningen, Netherlands) and involved 30 min of continuous cycling at an intensity equivalent to 50% of *W*_max_ with a 2-min warm up and cool down (25 W) as previously described (van Dijk et al. 2012).

### Calculations

The 24-h period of interest for continuous glucose measurements commenced at the start of the breakfast period on day 2. The continuous glucose data were exported to Excel (Microsoft, Washington, USA), the relevant 288, 5-min replicate values were isolated, and subsequently converted in summary variables including mean 24-h glucose (primary outcome), and secondary outcomes including glycaemic variability, proportion of time in hyperglycaemia, 24-h incremental (above fasting; calculated from the mean glucose 30 min prior to breakfast) AUC (iAUC), and the total AUC for 3 h breakfast, lunch and dinner responses. AUC was calculated using the trapezoid rule. The 3-h postprandial glucose (3-h PPG) was defined as mean glucose 150–180 min following each main meal. The glycaemic range, the mean amplitude of glycaemic excursions (MAGE) and the continuous overall net glycaemic action (CONGA) were calculated as measures of glycaemic variability (Rodbard 2009) using a freely available Excel Macro (Easy GV 9.0; available at http://www.easygv.co.uk). The hyperglycaemic threshold was defined as ≥ 9 mmol/l based on the published International Diabetes Federation criteria (International Diabetes Federation 2014).

For the analysis of the Actihearts, minute by minute energy expenditure was calculated using the manufacturers software (Actiheart, Cambridge Neurotechnology Ltd., Cambridge, UK) and subsequently exported to Microsoft Excel for determination of physical activity summary variables including total 24-h energy expenditure (TEE), physical activity level (PAL; total energy expenditure / resting energy expenditure), sedentary time (mins < 1.5 METs), time in light PA (mins > 1.5 METs but < 3 METs), time in moderate PA (mins > 3 METs and < 6 METs) and time in vigorous PA (mins > 6 METs) (Haskell et al. 2007; Pate et al. 2008).

### Statistical analysis

All statistical analysis was performed in GraphPad Prism 7 for Mac OS X. Differences between conditions for exercise responses (i.e. power output and exercise energy expenditure), as well as for 24-h CGM and PA summary variables, were compared using a one factor (condition) repeated measures ANOVA. ANOVA was performed regardless of any minor deviances from a normal distribution and with the Greenhouse–Geisser correction applied in cases of violated sphericity (Maxwell and Delaney 2004). If significant main effects were observed for 24-h CGM and PA variables, then to address our planned primary aim and hypothesis we compared each of the three exercise conditions to control with a paired t-test and a Bonferroni correction for multiple comparisons. For comparisons of exercise responses, the one-way ANOVA was followed up with Bonferroni-corrected *t* tests to locate differences between exercise conditions. Statistical significance was set at *p* ≤ 0.05 (two-tailed) and, unless stated otherwise, data are presented as mean ± SD. Cohens *d* was calculated as a measure of effect size with the following thresholds: small (*d* = 0.2), medium (*d* = 0.6) and large (*d* = 1.2) effect.

## Results

### Exercise characteristics/intervention fidelity

All participants successfully completed the three exercise sessions. During the exercise work intervals, mean power output was lowest during MICT (97 ± 17 W), higher during HIIT (165 ± 28 W, *p* < 0.05 vs MICT), and higher still following REHIT (417 ± 49 W, *p* < 0.05 vs HIIT and MICT). On the other hand, Actiheart-estimated energy expenditure during exercise was, on average, lowest during REHIT (251 ± 94 kJ), higher during HIIT (921 ± 279 kJ, *p* < 0.05 vs REHIT), and tended to be highest during MICT (1076 ± 378 kJ, *p* < 0.05 vs REHIT, *p* = 0.07 vs HIIT). During REHIT, peak, average and end power output were 5.9 ± 0.7, 4.8 ± 0.6 and 3.7 ± 0.6 W/kg for the first sprint, and 5.6 ± 0.5, 4.4 ± 0.5 and 3.2 ± 0.6 W/kg for the second sprint, respectively.

**Fig. 2:**
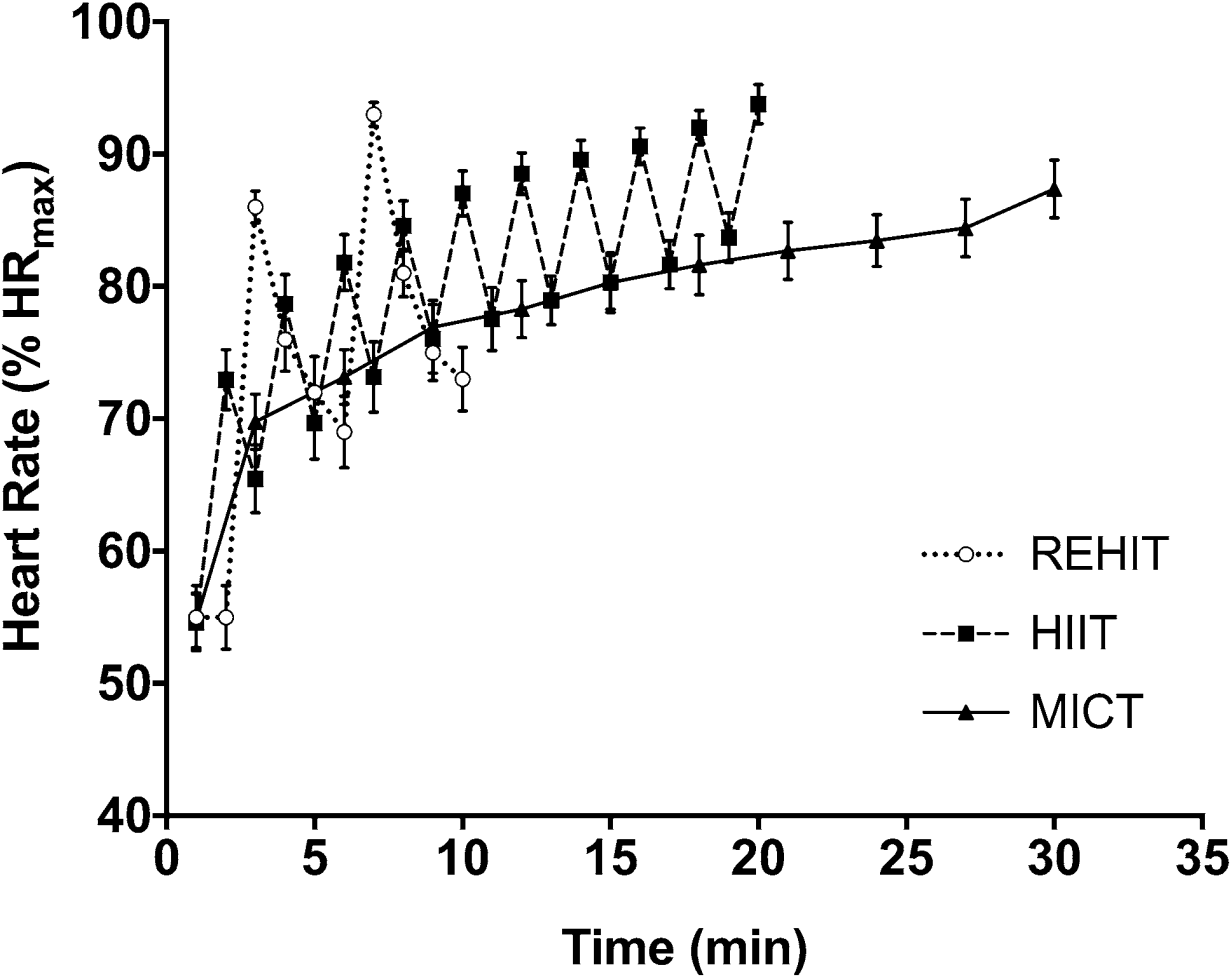
Heart rate responses over time with each exercise session. Data are presented as mean and SEM for visual clarity

MICT elicited a mean exercise heart rate of 80 ± 5% of the HR_max_ achieved during the *V*O_2peak_ test, whilst during the HIIT work intervals there was a progressive increase in heart rate, which reached 89 ± 5%, 90 ± 5%, 91 ± 5%, 92 ± 4% and 94 ± 5% of HR_max_ during intervals 6–10, respectively (Fig. 2). Heart rate during REHIT peaked at 86 ± 4% and 91 ± 3% of HR_max_ during sprint 1 and 2, respectively, but mean exercise heart rate during REHIT was 74 ± 12% of HR_max_ (Fig. 2).

The impact of the four-trial conditions on 24-h physical activity energy expenditure derived from the Actihearts is shown in Table 2. There were significant main effects of condition for all PA parameters (*p* < 0.05 for all). Both HIIT and MICT appeared to increase total 24-h energy expenditure (*p* < 0.05 for both), light PA (*p* < 0.05 for both), moderate PA (*p* = 0.06 for both), and decrease sedentary time (*p* < 0.05 for both), when compared with CON. Vigorous PA was significantly increased with HIIT compared with CON only (*p* < 0.05). Although 24-h TEE, light PA, moderate PA, and vigorous PA were higher, and sedentary time lower, on average with REHIT compared with CON, the differences were smaller and [with the exception of light PA (*p* < 0.05)] not statistically significant.

### Effects of exercise on glycaemic parameters

#### 24-h summary variables

**Fig. 3:**
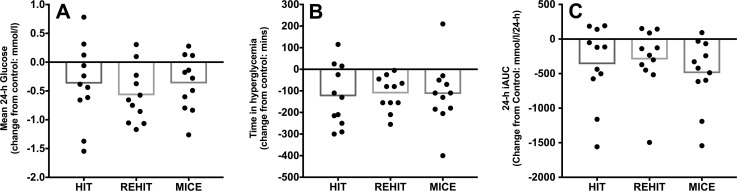
Effect of exercise on 24-h CGM summary variables. Data are presented as mean change (bars) with individual change scores (dots) compared with CON

The mean effect of exercise on 24-h glycaemic summary variables is shown in Table 4 with individual participant change scores (exercise minus control) for key summary variables shown in Fig. 3. There were significant effects of condition for mean 24-h glucose (*p* = 0.05), time in hyperglycaemia (*p* = 0.04) and for 24-h iAUC (*p* = 0.02). Mean 24-h glucose was lower during REHIT (*p* = 0.008, *d* = 0.55) and tended to be lower during MICT (*p* = 0.08, *d* = 0.35) when compared to CON, but there was no statistically significant change observed with HIIT (*p* = 0.31, *d* = 0.35). Time spent in hyperglycaemia appeared to be lower following all three exercise conditions compared with CON (REHIT: *p* = 0.002, *d* = 0.50; MICT: *p* = 0.08, *d* = 0.50; HIT: *p* = 0.04, *d* = 0.54), whilst 24-h iAUC was significantly reduced following MICT only (*p* = 0.02, *d* = 0.89). There were no significant changes in 24-h SD, glycaemic range, MAGE or CONGA with any of the exercise conditions (*p* > 0.05 for all main effects, respectively; Table 4).

**Table 4:** Effects of exercise compared with no-exercise control on CGM-derived glycaemic parameters

Variable	CON	ANOVA	Exercise condition					
			REHIT		MICT		HIIT	
	Mean ± SD	Main effect (*p*)	Mean ± SD	∆ (95%CI)	Mean ± SD	∆ (95%CI)	Mean ± SD	∆ (95%CI)
24-h mean glucose	8.11 ± 1.05	0.05	7.53 ± 0.87	− 0.58 (− 0.16, − 0.99)*	7.74 ± 1.10	− 0.37 (0.04, − 0.78)^†^	7.74 ± 0.59	− 0.37 (0.22, − 0.96)
24- h iAUC (mmol/L/24 h)	1340 ± 554	0.02	1044 ± 541	− 296 (102, − 694)	846 ± 516	− 494 (− 66, − 922)*	976 ± 406	− 364 (127, − 855)
Time ≥ 9 mmol/L (min)	353 ± 211	0.04	241 ± 190	− 112 (− 43, − 181)*	238 ± 287	− 115 (13, − 242)^†^	228 ± 133	− 125 (− 3, − 247)*
Glycaemic range	6.49 ± 2.91	0.96	6.29 ± 2.68	− 0.20 (1.87, − 2.27)	6.53 ± 3.12	0.03 (1.94, − 1.87)	6.39 ± 2.39	− 0.10 (1.72, − 1.92)
MAGE	4.21 ± 2.04	0.12	3.76 ± 1.35	− 0.45 (0.56, − 1.46)	3.47 ± 1.59	− 0.74 (0.36, − 1.84)	3.42 ± 1.50	− 0.79 (0.52, − 2.09)
CONGA	7.25 ± 1.00	0.06	6.69 ± 0.72	− 0.57 (0.03, − 1.11)	6.93 ± 0.93	− 0.33 (0.18, − 0.83)	6.89 ± 0.53	− 0.36 (0.26, − 0.99)
Peak glucose breakfast	11.86 ± 2.84	0.24	11.19 ± 2.61	− 0.67 (0.76, − 2.10)	12.10 ± 3.06	0.24 (1.68, − 1.21)	12.02 ± 2.467	0.15 (1.25, − 0.94)
Peak glucose lunch	9.63 ± 1.33	0.28	9.12 ± 1.26	− 0.51 (0.12, − 1.13)	9.19 ± 1.30	− 0.44 (0.22, − 1.09)	8.97 ± 0.84	− 0.65 (0.62, − 1.93)
Peak glucose dinner	10.00 ± 1.12	0.06	9.43 ± 1.34	− 0.57 (0.35, − 1.50)	9.10 ± 1.25	− 0.90 (0.03, − 1.83)	9.89 ± 1.16	− 0.11 (0.52, − 0.74)
3- h PPG breakfast	9.08 ± 3.10	0.64	9.63 ± 3.10	0.55 (1.66, − 0.55)	9.41 ± 3.48	0.33 (2.04, − 1.37)	9.11 ± 2.96	0.03 (1.76, − 1.69)
3-h PPG lunch	8.41 ± 1.10	0.36	7.99 ± 1.24	− 0.42 (0.56, − 1.39)	7.99 ± 1.28	− 0.41 (0.40, − 1.23)	7.77 ± 0.76	− 0.64 (0.57, − 1.85)
3-h PPG dinner	8.78 ± 1.38	0.06	7.79 ± 1.17	− 0.99 (− 0.19, − 1.79)	7.65 ± 1.00	− 1.13 (− 0.01, − 2.25)	8.05 ± 0.77	− 0.73 (0.72, − 2.18)

Note that all data are in mmol/L unless stated otherwise

*PPG* postprandial glucose, *AUC* area under the curve, **∆** exercise condition–control

*p < 0.05 vs CON

^†^ *p* = 0.08 vs CON

#### Meal responses

**Fig. 4:**
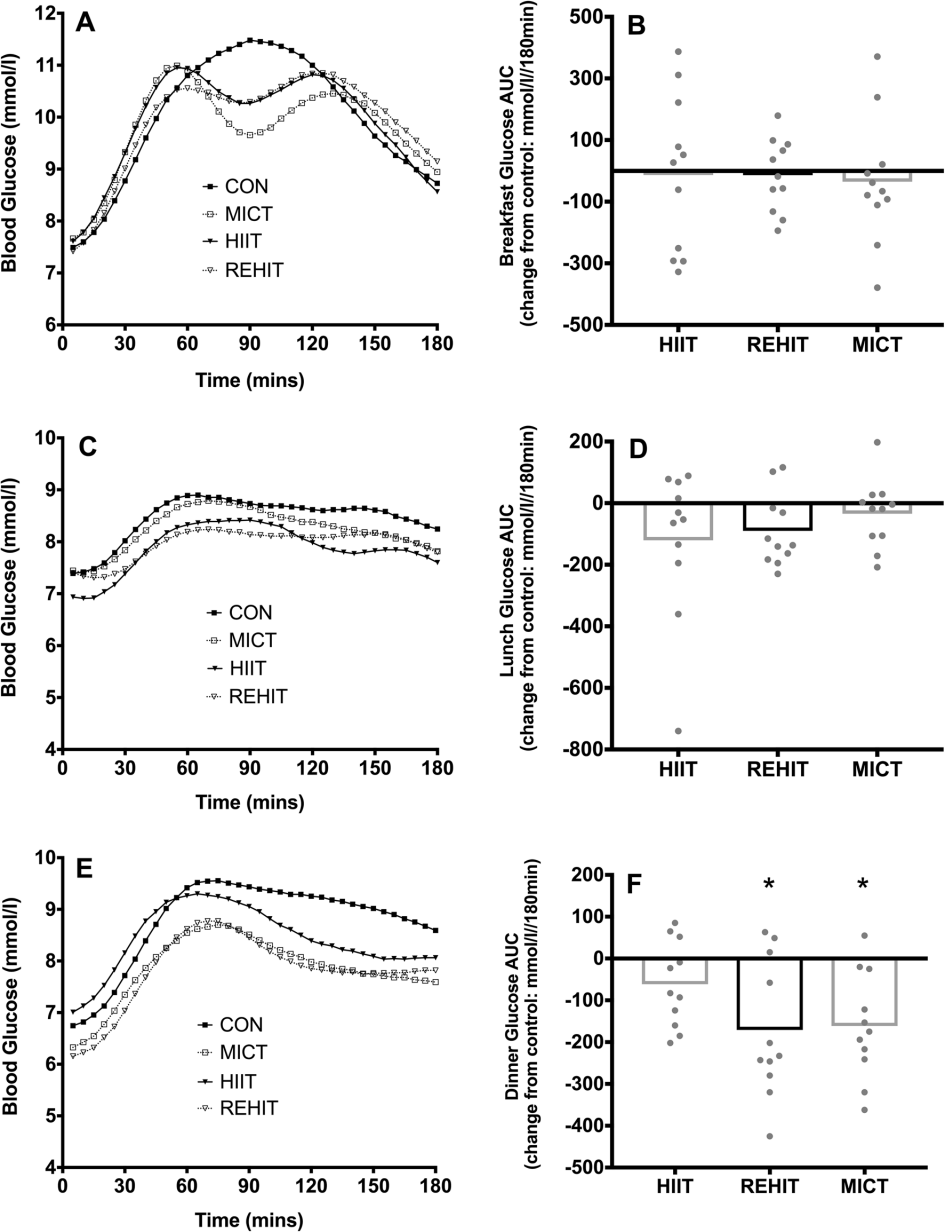
Glucose time responses (**a, c, e**) and AUC (**b, d, f**) for breakfast (**a, b**), lunch (**c, d**) and dinner (**e, f**). *Denotes *p* < 0.05 vs CON. Glucose time responses are presented as means only for clarity, whilst AUCs are presented as mean change (bars) with individual change scores (dots) compared with CON

The 3-h glycaemic responses to breakfast, lunch and dinner are shown in Fig. 4 with additional postprandial summary variables in Table 4. There were no differences between conditions in the glycaemic response to breakfast or lunch (both *p* > 0.05, respectively). However, there was a significant effect of condition on the AUC for dinner (*p* = 0.004), with lower AUC following REHIT (− 11%, *p* = 0.018, *d* = 1.05) and MICT (− 11%, *p* = 0.006, *d* = 0.99) compared with CON. The glycaemic response to dinner was not significantly affected by HIIT (−4%, *p* = 0.22, *d* = 0.38). There were no significant main effects of condition on any other postprandial variable (Table 4).

### Adverse events

One participant reported subjective symptoms of hypoglycaemia between lunch and dinner during the HIIT trial; however, glucose recorded via the CGM appeared to be within the normal range (between 5 and 6 mmol/l). There were no other adverse events.

## Discussion

This study examined the acute effects of three discordant exercise strategies, performed after breakfast, on CGM-derived 24-h glycaemic control in 11 men with type 2 diabetes. We replicate the findings of numerous previous studies that have shown the beneficial effects of a single 30-min bout of MICT on the 24-h glycaemic profile in type 2 diabetes (van Dijk et al. 2012, 2013), and provide the first evidence to suggest that a modified SIT protocol (REHIT), requiring 40 s of high-intensity exercise within a total time commitment of 10 min, is also associated with positive glycaemic effects in the post-exercise period. This finding is both novel and of potential significance, as it provides the first evidence for a genuinely time-efficient exercise option to improve glycaemic control in individuals with type 2 diabetes who currently perceive lack of time as a barrier to performing regular structured exercise (Korkiakangas et al. 2009).

REHIT was associated with small (Cohens *d* between 0.2 and 0.6) but significant beneficial decreases in 24-h mean glucose and the prevalence of hyperglycaemia relative to a no-exercise control trial. This appears to have been predominantly driven by a marked reduction in the glycaemic response to the evening meal, as the breakfast and lunch responses were not significantly affected. Importantly, the improvements in glycaemic control were observed in addition to the impact of participants’ current medication, given the response to the no exercise (i.e. medication only) control trial. It is well established that hyperglycaemia is associated with endothelial cell stress and subsequent vascular dysfunction (Paneni et al. 2013), whilst improving glycaemic control reduces the risk of microvascular complications in type 2 diabetes (Stolar 2010). This is largely based on analysis of fasting glucose concentrations, OGTT glucose responses, or HbA_1c_, as estimates of glycaemic control, so it is difficult to draw direct comparisons on relative risk reduction for microvascular complications based on CGM variables (Monnier and Colette 2015). For example, a reduction in mean 24-h glucose can reflect changes during both ‘ambient’ and ‘postprandial’ periods (Monnier and Colette 2015). Nevertheless, it is reasonable to suggest that the ~ 0.5 mmol/l average reduction in 24-h mean glucose and ~ 2-h reduction in the prevalence of postprandial hyperglycaemia (per day) observed with REHIT, when performed on a regular basis, would make a meaningful impact on overall glycaemia (i.e. HbA_1c_) and hence long-term risk (Monnier and Colette 2015). The lower glucose AUC (− 11%) observed following dinner further supports this assertion, given that the relative contribution of postprandial hyperglycaemia to overall glycaemic exposure is greater in patients with HbA_1c_ ≤ 7.3% (Monnier and Colette 2015).

There is also a strong correlation between postprandial hyperglycaemia and the risk of adverse cardiovascular events (Coutinho et al. 1999). However, whether intervening to improve glycaemic control lowers cardiovascular risk over the long term is currently contentious (Wing et al. 2013). Nonetheless, combined with evidence that REHIT improves cardiorespiratory fitness and resting blood pressure with 8 weeks of thrice weekly training sessions (Ruffino et al. 2017), the current study provides further (tentative) evidence that REHIT favourably modifies the cardiovascular risk profile in those with type 2 diabetes. The lack of improvement in insulin sensitivity and glycaemic control reported 3 days after the final training session by Ruffino et al. (2017) suggests that the positive acute effects on glycaemic control are short lived. Future research should determine the optimal frequency of REHIT to maintain the acute benefits on glycaemic control.

The fact we could largely replicate the findings of previous studies on MICT and glycaemic control (van Dijk et al. 2012, 2013) validates our methodology and strengthens these preliminary findings with REHIT. However, it should be highlighted that the magnitude of the effects observed with both MICT and REHIT in the present study are smaller than in previous studies (van Dijk et al. 2012, 2013). We suspect that this is explained by the fact that our participants’ type 2 diabetes was relatively well controlled according to HbA_1c_. Van Dijk et al. (2013) demonstrated that reductions in, for example, mean 24-h glucose were lower (− 0.6 mmol/l vs − 1.2 mmol/l) in well controlled (HbA_1c_ < 7.0%) compared with sub-optimally controlled (HbA_1c_ > 7%) individuals with type 2 diabetes, respectively. The mean reductions in 24-h glucose of ~ 0.6 mmol/l with REHIT and ~ 0.4 mmol/l with MICT in the present study are, therefore, in line with the literature (Van Dijk et al. 2013).

The lack of statistically significant improvement in most glycaemic parameters with HIIT is an unexpected finding, particularly given the improvements observed with REHIT. Gillen et al. (2012) used a comparable HIIT protocol, trial design, and participants (*n* = 7) of similar diabetic status, and reported that HIIT markedly lowered average postmeal glucose spikes, as well as the glucose concentrations 60–120 min after the three post-exercise meals. In contrast, we observed no significant change in the AUC to any post-exercise meal or in any other marker of postprandial glycaemia. Similar, however, was the lower prevalence of hyperglycaemia despite no significant change in mean 24-h glucose (Gillen et al. 2012). It is important to highlight that the mean change for several of the glycaemic variables assessed with HIIT were in a favourable direction in the current study, but there was greater variation in individual change scores than with REHIT and MICT. It is possible that with an increased sample size and greater statistical power we would have observed effects of HIIT on other glycaemic parameters. Considering the accumulating evidence for beneficial training effects with this HIIT protocol in type 2 diabetes (Little et al. 2011; Francois et al. 2017), we would encourage larger definitive studies of the acute effects on glycaemic control.

There are several considerations in the study design and employed techniques that should be acknowledged. First, although we provided standardised food packages during all trials, and asked participants to record the time of their medication, the study was otherwise free-living and (although this is also a strength of the study) we only have participants’ self-reported compliance. In addition, although CGM captures glycaemic data outside the laboratory and has shown good agreement with responses measured simultaneously in venous blood (Bailey et al. 2014), the coefficient of variation for some parameters can be high (Terada et al. 2014). The fact we could detect differences despite this lower level of control is encouraging, but we acknowledge that our small sample size, although consistent with numerous other studies on this topic (van Dijk et al. 2012, 2013; Manders et al. 2010), is a key limitation and our data should be interpreted with caution in that context. Larger free-living CGM studies combined with more controlled laboratory assessments will be required to confirm our preliminary findings. We were also only able to capture postprandial glucose responses in this study and can provide no mechanistic insight, and so future investigations should aim to provide a more holistic and mechanistic assessment of carbohydrate and lipid metabolism following REHIT in both men and women with type 2 diabetes.

In conclusion, this study suggests that a brief bout of REHIT improves markers of postprandial glycaemic control over the following 24-h period when compared with no exercise. We conclude that REHIT may offer a genuinely time-efficient exercise option for men with type 2 diabetes and warrants further study.

## Data Availability

The raw data for this study are available from the corresponding author on reasonable request.

## Abbreviations

ADA: American Diabetes Association
AUC: Area under the curve
iAUC: Incremental area under the curve
BMI: Body mass index
CGM: Continuous glucose monitoring
CONGA: Continuous net overlapping glucose action
HbA_1c_: Glycated hemoglobin
HIIT: High-intensity interval training
HR: Heart rate
HRmax: Maximal heart rate
MAGE: Mean amplitude of glucose excursions
MICT: Moderate-intensity continuous training
PA: Physical activity
PAL: Physical activity level
REHIT: Reduced-exertion high-intensity interval training
SIT: Sprint interval training
*V*O_2_: Oxygen uptake
*V*O_2peak_: Peak oxygen uptake
*W*_max_: Peak power output
